# Action Observation and Motor Imagery in Children with Developmental Coordination Disorder: A Systematic Review

**DOI:** 10.3390/brainsci16020234

**Published:** 2026-02-17

**Authors:** Elisa De Masi, Giovanni Morone, Giorgia Bruschi, Maria Paola Colatei, Martina D’Arienzo, Giulia Pezzetta, Irene Ciancarelli, Alex Martino Cinnera

**Affiliations:** 1 Scientific Institute for Research, Hospitalization and Health Care IRCCS Santa Lucia Foundation, 00179 Rome, Italy; elisadm32@gmail.com (E.D.M.); m.darienzo@hsantalucia.it (M.D.); a.martino@hsantalucia.it (A.M.C.); 2 Department of Life, Health and Environmental Sciences, University of L’Aquila, 67100 Aquila, Italy; mariapaola.colatei@univaq.it (M.P.C.); irene.ciancarelli@univaq.it (I.C.); 3 Neuro-and-Psychomotor Therapy for Developmental Age, University of L’Aquila, 67100 Aquila, Italy; giorgia.bruschi@student.univaq.it (G.B.); giulia.pezzetta@student.univaq.it (G.P.)

**Keywords:** motor skill disorders, children with disabilities, internal modeling deficit, mental training, motor planning, motor simulation techniques

## Abstract

Background and Objectives: Children with Developmental Coordination Disorder (DCD) show substantial motor and balance difficulties that affect daily activities. Although action observation (AO) and motor imagery (MI) are effective in other neurological conditions, their impact in DCD remains underinvestigated. This review explores the preliminary evidence of AO- and MI-based interventions for improving motor and functional outcomes in children with DCD. Methods: A systematic search of PubMed, Scopus, and Web of Science identified randomized controlled trials and controlled trials published in the last 15 years evaluating AO and MI interventions in children with DCD. Two independent reviewers conducted the screening of the studies, data extraction, and the risk-of-bias assessment using RoB2 and ROBINS-I. The review followed PRISMA reporting guidelines and was pre-registered on the PROSPERO database (CRD420251084196). Results: Of 320 records initially identified, seven studies, involving 199 children with DCD (aged 5–12 years), were included. Interventions varied from single-session to multi-session protocols (1–16 sessions) and included AO, MI, or a combination of both (AO + MI), with heterogeneous control conditions. Within these studies, the outcomes were primarily assessed using standardized motor coordination measures (MABC/MABC-2, DCDQ), planning tasks, and performance-based activities of daily living (ADLs) measures. Improvements were reported in motor imagery tasks, planning, and functional task performance. However, RCTs and CTs were identified to have a moderate and high risk of bias, respectively. Conclusions: The present review suggests that AO and MI, either alone or in combination, may enhance motor planning, coordination, and daily functional skills in children with DCD, supporting internal motor representations and predictive motor control, reflecting functional gain in motor skills and ADL performance. Interestingly, these mental training approaches can be applied in clinical and everyday settings and are suitable for supporting these processes, with VR-based combinations representing a promising, but exploratory, approach. Although critical heterogeneity and a moderate risk of bias remain, the findings need to be interpreted with caution and require further investigation.

## 1. Introduction

Children diagnosed with Developmental Coordination Disorder (DCD) exhibit difficulties in acquiring age-appropriate motor skills, despite the absence of any general medical condition, pervasive developmental disorder, or intellectual disability [1]. These motor deficits substantially interfere with the activities of daily living (ADLs) such as hygiene, schoolwork, social participation, and sports [2]. A primary motor control issue in DCD is impaired balance, with reports indicating that up to 87% of affected children experience balance difficulties [3,4]. Motor deficits are often also associated with deficits in motor imagery (MI) in children with DCD [5], especially in complex task constraints [6,7]. MI, which involves the mental simulation of a movement without any physical execution or muscle activation, and action observation (AO), which entails watching another individual perform a movement, both engage brain regions similar to those activated during the actual movement and may play an equivalent role in motor learning [8]. Mirror neurons, first identified in the premotor cortex of monkeys [9], are thought to support a mirror mechanism that maps observed actions onto the neural substrates responsible for their execution [10]. This mechanism enables an experiential understanding of others’ actions through the reactivation of motor representations, a process that also occurs during MI, when movements are mentally reproduced. Increasing neuroscientific evidence suggests that motor simulation techniques share this neural basis and contribute to motor learning. Based on neuroscientific evidence, AO and MI have been widely used as therapeutic interventions to address motor impairments across a range of neurological disorders, with generally positive effects reported. Indeed, recent literature reviews consistently highlight the positive effects of these rehabilitative approaches on motor and functional outcomes, particularly in populations affected by Parkinson’s disease and stroke [11,12,13,14]. Although children with DCD have been shown to present alterations in MI abilities, particularly under complex task constraints, and despite the well-documented effectiveness of AO- and MI-based interventions in other neurological disorders, no systematic reviews have yet evaluated their efficacy as rehabilitative approaches in this population. In fact, current evidence remains limited, with small sample sizes and substantial heterogeneity in intervention protocols and study procedures. The aim of the present systematic review is to explore and summarize the latest findings regarding the use of AO and MI, or their combination, in the treatment of motor, balance, or functional impairment in children diagnosed with DCD.

## 2. Materials and Methods

To explore the most recent evidence on the effects of AO and MI training on motor functions in children with DCD, we conducted a systematic search of articles indexed in PubMed, Scopus, and Web of Science (WOS) published over the past 15 years, a time frame chosen to reflect methodological and technological advancements in mental training for this pediatric population (search strategy is available in Table S1). Studies were selected based on the following criteria: (1) participants under 18 years of age; (2) a clinical diagnosis of DCD; (3) intervention based on AO and/or MI therapy; (4) randomized controlled trials (RCTs) or controlled trials (CTs); (5) studies published in English to ensure a wide range of high-impact international scientific literature.

Exclusion criteria were as follows: (1) studies involving adult participants or mixed-age samples without separate pediatric data; (2) studies in which AO or MI was not the primary intervention; (3) non-interventional study designs (e.g., observational studies, case reports, reviews, or expert opinions); (4) articles not available in full text; and (5) articles not published in peer-reviewed journals. All retrieved articles were imported into an online database [15] and independently screened by two reviewers based on title and abstract. Discrepancies were resolved through discussion with a third reviewer to achieve consensus. The same procedure was applied during the full-text evaluation. The reporting followed the PRISMA statement. The protocol was pre-registered on PROSPERO database (CRD420251084196) on 21 January 2025.

### 2.1. Data Extraction

The data from the selected studies were independently extracted by two authors and organized to provide a clear and structured synthesis of the available evidence. The extracted information was grouped into several key categories. Bibliometric data included the author(s), year of publication, and country of origin. Methodological characteristics comprised the study design and its corresponding level of evidence. Details about the study population were also recorded, including sample size, sex distribution, and participants’ age range. Information related to the intervention covered the duration and nature of the treatment, as well as the composition of both experimental and control groups. Inclusion and exclusion criteria were noted to better understand the population under investigation. Furthermore, details regarding the measurement tools and the timing of outcome evaluations were collected. The measures of effect were reported using statistical significance (*p*-values) and, when available, effect sizes, including Cohen’s d, r, or η^2^, depending on the metrics provided in the original studies. Finally, the main findings and conclusions reported by the authors were summarized. This comprehensive and structured approach allowed for a thorough overview of each study, facilitating comparison across trials and supporting a critical appraisal of the current body of evidence.

### 2.2. Risk of Bias Assessment

The risk of bias of included studies was assessed using the Cochrane risk of bias tool version 2 (RoB2) and the Risk of Bias in Non-randomized Studies of Interventions (ROBINS-I), for RCT and CT, respectively, by two blinded assessors. The overall risk of bias (RoB) for each study was categorized as low, moderate, or high quality based on the number and severity of the identified biases across relevant domains. Studies were classified as low risk (high quality) if all domains were judged to have a low risk of bias; as moderate risk (moderate quality) if at least one domain had unclear or moderate risk but none were high; and as high risk (low quality) if one or more domains were deemed to have a high risk of bias.

## 3. Results

After the initial search, 320 records were identified. Following the removal of 89 duplicates, 231 articles were screened by title and abstract. Of these, 223 underwent full-text assessment, and ultimately 7 studies met the inclusion criteria [16,17,18,19,20,21,22] (Figure S1). The included studies were published between 2016 [16] and 2023 [22]. Most studies were conducted in the United Kingdom [20,21,22] and Australia [16,18], of which four are RCTs [16,19,20,22] and three are CTs [17,18,21]. Marked heterogeneity was observed across the included studies, arising from differences in participant characteristics (confirmed versus suspected DCD), intervention dosage (single-session versus cumulative training), control conditions (active, passive, or within-group comparisons), and the use of both proximal and distal outcome measures to assess intervention effects.

### 3.1. Population

The included studies enrolled a total of 199 (28.4 ± 12.1) children diagnosed with DCD, ranging from a minimum of 8 patients [17] to a maximum of 42 [16]. The included studies involved children with an age range from 5 to 12 years old. In the studies that reported sex among included children [17,18,19,20,22] a balanced distribution was observed (male 52.9%; female 47.1%). All participants met the DSM-5 diagnostic criteria for DCD; two of the included studies also recruited in their trial children with suspected DCD, who were screened before they started the training using measurement tools such as MABC-2 (Movement Assessment Battery for Children Second Edition) and DCDQ (Developmental Coordination Disorder questionnaire) specifically made for assessing children with perturbations of the motor skills or coordination in early ages, before the diagnostic process [20,22]. Yet these children present the same deficits as children with DCD diagnosis, so the choice to include them, at an early stage of the development, into a targeted trial is decisive to prevent or reduce the development of more advanced symptoms (detailed information of included studies is reported in Table 1).

### 3.2. Intervention

Three studies implemented a single-session intervention in which children completed the trials in predefined blocks [18,20,21]. The remaining studies delivered training over multiple sessions, with frequencies ranging from once per week [16,17] to four times per week [22]. Overall, the total number of sessions varied considerably, from a single session [18,20,21] to sixteen sessions [19,22], with an average of 6.3 ± 6.8 sessions. In studies with multiple sessions, the total study duration ranged from four weeks [22] to nine weeks [17], with a mean duration of 4.1 ± 3.4 weeks across studies [17,19,22]. Among the four studies clearly reporting session length, AO + MI activities lasted between 10 min and 1 h. Across the included studies, MI training typically involved guided internal rehearsal of both functional and sport-related movements. Participants engaged in kinesthetic or visual imagery scripts tailored to everyday tasks (such as shoelace tying, cutlery use, shirt buttoning, and cup stacking) [22] or to broader fundamental motor skills including catching and throwing a tennis ball, striking a softball, jumping to a target, balancing a ball while walking, and placing objects on a form board [16]. In several cases, MI was combined with periods of overt practice to help refine internal motor representations. AO and combined AO + MI approaches used video demonstrations to support motor learning; participants viewed examples of conventional performance strategies [22] or observed first-person videos showing the progression of a novice learning a visual-motor rotation task [20] while simultaneously generating kinesthetic imagery. In AO + MI protocols, observation was systematically followed by physical execution (for example, guiding a stylus-controlled cursor to sequential visual targets) to promote continuous updating of the internal model. Some studies implemented immersive game-based or virtual reality training (VR training) [19], where participants selected interactive games (e.g., mini-baseball, basketball, bowling, soccer) designed to challenge object-control and manipulative skills including throwing, catching, dribbling, kicking, and striking. A structured, multicomponent format was adopted in Adams and colleagues (2017) [17], where each session combined goal setting, third- and first-person action observation, mental rehearsal, overt practice, and alternation between imagery and execution with guided reflection. Specific object-manipulation paradigms were also used [18]; participants grasped and rotated an octagonal dial to match color sequences, either performing the movements directly or first imagining how they would grasp and rotate the object before acting. Finally, Scott and colleagues [21] employed an imitation-based approach in which participants viewed a brief static image followed by a video of a rhythmic action and then reproduced the movement while 3-D kinematics were recorded. Across the included studies, most interventions were preceded by a pre-test phase used to familiarize participants with the task demands and to establish a baseline level of performance. These pre-tests typically involved brief demonstrations, exposure to the task environment, or completion of initial trials to ensure that subsequent training effects could be interpreted relative to a clearly defined starting point.

### 3.3. Control

Across the selected studies, several control conditions were used, including no intervention, typically developing controls, and alternative rehabilitative approaches. Other studies used active but non-specific controls, for example, children played games that were unrelated to the targeted ADLs to match engagement and screen time without training the target skills [22]. A commonly used sensory-control was viewing neutral video content [20]; participants watched clips of a nature documentary that contained no human motor content, with clip duration chosen so total viewing time equaled that of the AO + MI videos. Finally, some studies compared interventions to structured alternatives such as CO-OP (Cognitive orientation to daily occupational performance; goal-directed training), which typically included goal discussion and planning, practice using the Goal-Plan-Do-Check framework, and homework/parent guidance [17]. Several studies therefore balanced time, attention, and physical practice across groups to isolate the specific effects of AO, MI, or AO + MI.

### 3.4. Outcome

Assessment tools encompass a range of proximal and distal motor outcomes, which can be mapped on key IMD processes (i.e., predictive control, forward modeling, motor planning, and end-state comfort). Standardized tests such as the MABC and MABC-2, as well as the DCDQ, were most frequently used (Table 2). The MABC/MABC-2 evaluates global motor coordination, including manual dexterity, reaching/grasping, and balance, representing distal functional outcomes, whereas the DCDQ, a parent-report questionnaire, served both as a screening tool for motor difficulties and for group allocation. Several studies employed tasks specifically targeting proximal IMD-related processes such as motor imagery and action planning. More specifically, these processes included the hand rotation task to assess mental rotation and internal action representation; anticipatory action planning tasks to evaluate predictive control and to sequence with respect to end-state comfort; and rapid online control tasks to measure forward modeling and adaptive corrections during goal-directed actions. Performance-based measures further included assessments of ADLs, evaluating both execution quality and movement technique, as well as completion time and target-locking scores in visuomotor tasks, thereby bridging proximal IMD processes and distal functional skills. Some studies additionally analyzed movement kinematics using three-dimensional motion capture to quantify spatial and temporal parameters of movement, offering more accurate insights into underlying control mechanisms. Complementary instruments, such as the MIQ-C and MCQ, assessed imagery ability and perceived motor competence, while the NDI evaluated neuromuscular development in younger children. Together, these measures allowed a comprehensive evaluation of motor function, spanning internal representations, predictive planning, and forward control mechanisms through to overall coordination and performance in everyday activities.

### 3.5. Evidence Synthesis

Across multi-session intervention studies [16,17,19,22] (three RCTs and one CT), statistically significant improvements were observed on a range of motor outcomes compared to control or baseline conditions. In standardized motor assessments, intervention groups showed greater pre–post gains compared to controls (*p* = 0.03; r = 0.70–0.84), corresponding to large effect sizes, but with no significant differences between active interventions (*p* = 0.95) [16]. Similarly, significant group × time interactions were found for MI (*p* = 0.039, η^2^ = 0.16) and action planning (*p* = 0.027, η^2^ = 0.17), with the experimental group outperforming the control group at post-test and follow-up. Significant interaction effects were also reported for online anticipatory action control, total time on target (*p* < 0.001, η^2^ = 0.47), consecutive time on target (*p* < 0.001, η^2^ = 0.44), and distance from target (*p* = 0.001, η^2^ = 0.22), indicating improvements in the experimental group from pre- to post-test that were maintained at follow-up [19]. Significant group × time interactions, pre–post comparison and retention were observed for functional motor tasks, including shoelace tying (*p* = 0.018; post-test *p* = 0.045, technique *p* = 0.002, retention *p* = 0.011), and cup stacking (post-test *p* = 0.008, retention *p* = 0.01) [22]. Within-group significant results were also observed in the shirt buttoning (post-test: *p* = 0.002, retention: *p* = 0.001), and cutlery use (post-test: *p* = 0.026, retention: *p* = 0.001). Finally, based on descriptive statistics of Adams and colleagues (2017) [17], clinically meaningful improvements were also observed on the MABC-2 scores in both the experimental and active control groups.

Across single-session imagery-based investigations [18,20,21], significant improvements were reported on various motor and predictive control outcomes. Increases in movement efficiency and reductions in suboptimal strategies were observed (*p* ≤ 0.014) [18]. Significant group × time interactions were observed for completion time (*p* = 0.009) and target-locking scores (*p* ≤ 0.012), confirming better post-test performance in the AO-MI group compared with control group [20]. Finally, imitation performance improved following combined imagery-based instructions compared with motor imagery alone (*p* = 0.021). However, differences relative to simple imitation in children with DCD were not consistently significant (*p* = 0.236) [21].

### 3.6. Methodological Reporting Adherence

**Figure 1:**
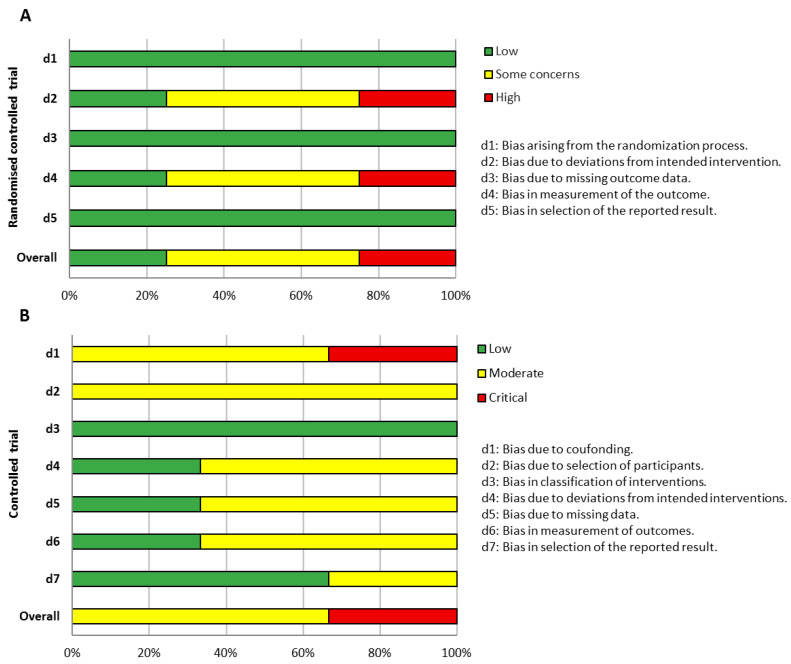
Bar plots illustrating the Risk of Bias domain assessments for included randomized controlled trials (RCTs; panel **A**) evaluated with the ROB 2 tool, and controlled trials (CTs; panel **B**) evaluated with the ROBINS-I tool.

In the assessment of reporting quality, adherence to the intervention and the measurement of variability in participant outcomes were the domains most frequently identified in the RCTs (Figure 1A). Item-level analysis indicated that these aspects were commonly related to the allocation to the experimental group and to the implementation of blinding procedures, reflecting characteristics of the experimental designs employed. Notably, a high risk of bias in these domains suggests that deviations from intended interventions and potential bias in outcome assessment may have influenced the results, limiting confidence in the observed effects and supporting a cautious interpretation of the findings.

For the CT studies, most investigations were classified as having an elevated overall risk of bias (Figure 1B). Across the included studies, there was limited control of confounding factors, such as age, severity of DCD, and comorbidities, and variability in adherence to the intervention and heterogeneity in outcome measures were observed. These issues reflect the lack of randomization and differences in participant selection procedures, which may have led to an overestimation of the reported intervention effects.

## 4. Discussion

The present systematic review explored the preliminary evidence of the effects of AO and MI therapies in the improvement of motor functions in children with DCD. The results suggest that mental training, particularly MI and its combination with AO, may be feasible and potentially beneficial for enhancing motor planning, coordination, and execution in ADL compared with control conditions. However, the heterogeneity across experimental procedures and the potential risk of bias impose caution when interpreting the magnitude of effects. Moreover, compared to active control interventions, effects were generally not statistically significant, suggesting that these approaches may be considered as add-on treatments.

Nevertheless, while preliminary and heterogeneous, the results suggest that motor simulation techniques may contribute to reducing motor control and coordination difficulties by engaging mechanisms potentially related to the mirror neuron system, thereby facilitating the reactivation of motor representations that could partially compensate for difficulties in generating internal forward models during physical execution.

However, these findings should be interpreted with caution. Although the findings are broadly consistent with the IMD framework, suggesting that mental training may influence aspects of predictive motor control in children with DCD [16,20], the current evidence does not support causal inferences, and the IMD rationale should be regarded as a plausible interpretative model rather than a definitively established explanatory mechanism. MI alone can support MP by encouraging children to use the end-state-comfort strategy more and enhance their motor skills, as shown in some included studies [16,18]. Indeed, when combining AO and MI, the benefits seem to increase, not only in the imitation of everyday actions (e.g., in the ADLs) [21,22] but also in adapting the visual system and neuro-motor system during complex tasks [20]. Therefore, combining AO and MI appears to offer potential benefits beyond those of single modalities (AO or MI). In fact, the simultaneous observation of an external guide to the internal kinesthetic simulation leads to a more robust internal model and to an improvement of the eye–hand coordination. The studies included in this review support the hypothesis that DCD rehabilitation should extend beyond physical practice to incorporate strategies aimed at strengthening internal motor representations and addressing deficits in internal modeling. Nevertheless, evidence on the combined use of AO and MI remains speculative due to the limited number of direct comparisons. Regarding distal outcomes, the most positive effects reported were observed through standardized measurement tools such as the MABC (or MABC-2) and DCDQ, which assess motor skills while providing a practical, everyday perspective on children’s difficulties in their environmental context. Other improvements were also registered through less standardized tools such as general motor performances in everyday tasks (ADLs) and imitation skills [17,21,22].

The present review suggests that AO and MI interventions in addition to conventional training are feasible and they might be delivered in various settings: clinical (inpatient, outpatient) and environmental (e.g., school, home) with a caregiver [17,19]. Interestingly, AO and MI might be delivered through virtual reality scenarios or exergaming, offering the possibility to increase the engagement and the participation [23]. Although explorative, the use of virtual reality can provide augmented sensory feedback (which plays a key role in functional changes associated with neuroplasticity) [24], and enhances the embodiment and allocentric perception of movement in children affected by neurodevelopmental disorders [25].

### Limitations

A major limiting factor in the studies here reviewed is the small sample size, with only three adopting active control conditions, which ensured better comparability. The use of mixed samples, with unclear proportions of formally diagnosed children in some studies, also introduces diagnostic uncertainty that may affect internal validity and limit generalizability [20,22]. Moreover, the effects of studies implementing a single training session were combined with those using cumulative interventions, and in studies including typically developing children as a control group, effects were primarily estimated from within-group changes. In studies with multiple experimental groups, changes in the AO and MI groups were primarily compared with control groups rather than with other experimental conditions. Outcomes combined both proximal measures (e.g., motor planning, imagery, task performance) and distal measures (e.g., overall coordination, ADLs), which may affect the distinction between direct intervention and broader functional effects. Moreover, the inclusion of different study designs (RCTs and CTs) further limits confidence in causal inferences and the robustness of the observed effects, thereby weakening the strength of the conclusions. Overall, the risk of bias across studies was moderate-to-high, mainly due to outcome measurement, deviations from intended interventions, confounding, and participant selection. Future research should address these issues through blinded assessments, stricter intervention adherence, improved control of confounders, and clearer participant selection criteria. Finally, gray literature and unpublished studies were not included in the present review, which may increase the risk of publication bias, as studies with non-significant or negative results are less likely to be published, potentially leading to an overestimation of the reported intervention effects.

## 5. Conclusions

This systematic review indicates that AO and MI, whether applied individually or in combination, are feasible and may support motor planning, coordination, and execution in children with DCD. Their combination appears to offer a complementary approach that could further facilitate recovery by enhancing imitation, visuomotor integration, and eye–hand coordination. These interventions can be implemented in clinical or naturalistic settings and may be integrated with other technologies, including virtual reality, to promote improvements in both proximal and distal outcomes, such as motor skills and activities of daily living (ADL). However, evidence remains limited and heterogeneous. Small sample sizes, varying intervention designs, and the high risk of bias can reduce confidence in the results. Consequently, while motor simulation techniques appear promising as adjunctive interventions, current evidence is still preliminary. Further trials are required to support their effectiveness and to clarify optimal delivery strategies.

## Figures and Tables

**Table 1:** Demographic characteristics and protocols.

Author (Year) Country [Cit.]	Study Design (Level of Evidence)	Sample Size [Drop-Out] (Sex) [Years Range]	Inclusion and Exclusion Criteria	Intervention Duration (Frequency) [Session Duration]	Experimental Protocol (Sample Size) [Setting]	Control Group (Sample Size)
Wilson (2016) Australia [16]	RCT (2)	42 [6] (NCR) [7,8,9,10,11,12]	Inclusion: DCD diagnosis. Exclusion: current or past history of neurological disease including head injury, psychiatric disorders or attention deficit disorder.	5 weeks (once/week) [60 min]	MI: visual imagery, relaxation protocol, visual modeling, mental rehearsal of skills from an external/internal perspective, overt practice. (12) [School environment]	Perceptual Motor Training (PMT): Gross-, fine-, and perceptual-motor activities, static and dynamic balance activities. (13) Ctrl group (11)
Adams (2017) Netherlands [17]	CT (3)	8 [none] (3M, 5F) [7,8,9,10,11,12]	Inclusions: four DSM-5 diagnostic criteria for DCD (MABC-2, DCDQ, onset symptoms, no other medical condition). Exclusion: other medical conditions that could cause motor impairment.	9 weeks (1/week + 4/week) ** [45 min + 10 min]	AO + MI 5 parts: - Discuss homework completed - Watch video selecting motor skill -Overt practice - alternate mental rehearsal -New homework (4) [School environment]	CO-OP 3 parts: -Discuss homework -Practice the selected motor skill using the Goal-Plan-Do-Check framework -New homework (4)
Bhoyroo (2019) Australia [18]	CT (3)	36 [4] (36 M) [7,8,9,10,11,12]	DCD Inclusion: DCD diagnosis with DSM-5 DCD Exclusion: ADHD diagnosis or any other neurological condition. TD Inclusion: A cut-off NDI equal or above 90. TD Exclusion: no diagnosed movement difficulties or neurological conditions. no concerns regarding their academic performance or learning ability.	Single session [NCR]	2 conditions: MP (Motor Planning) and MIP (Motor Imagery and Planning). (14) [Clinical setting]	TD children, same intervention as experimental group. (18)
EbrahimiSani (2020) Iran [19]	RCT (2)	40 [none] (40F) [7,8,9,10]	Inclusion: DCD diagnosis with DSM-5, BOTMP-2 percentile score <16th, PMOQ-T. Exclusion: comorbidities of movement and physical or neurological disorders, and intellectual disabilities.	8 weeks (2 times/week) [30 min]	VR intervention with exergaming on the functions of MI, action planning, and online control for internal and predicting modeling and ending with balance. (20) [School environment]	No intervention. (20)
Marshall (2020) UK [20]	RCT (2)	20 [none] (13M, 7F) [7,8,9,10,11]	Inclusion: confirmed or suspected DCD diagnosis. Exclusion: comorbidities known to affect sensorimotor function and had no diagnosis of learning difficulties or ADHD.	Single session [NCR]	AO + MI: improving speed and accuracy in a visual-motor performance after watching (AO) and reading the script (MI) about the task. Following each AO + MI trial, participants immediately performed a physical practice trial. (10) [Clinical setting]	Same intervention, watching instead a video not related to the task. (10)
Scott (2020) UK [21]	CT (3)	25 [none] (10M, 15F) [7,8,9,10,11]	DCD Inclusion: DCD diagnosis with DSM-5, ≤16th percentile in the MABC-2 Scale, IQ of >70. DCD Exclusion: IQ < 70 no vision impairment or physical injury, any diagnosis of learning disorders. TD Inclusion: ≥20th percentile in the MABC-2 Scale. TD Exclusion: no diagnosed movement difficulties or neurological conditions.	Single session [NCR]	AO + MI: Observed or imagined rhythmic actions (slow or fast), then reproduced them as accurately as possible. (13) [Clinical setting]	TD children, same intervention as exp group. (12)
Scott (2023) UK [22]	RCT (2)	28 * [none] (21 M, 7F) [7,8,9,10,11,12]	Inclusion: confirmed or suspected DCD diagnosis. Exclusion: any co-occurring medical condition known to further impair learning or motor function learning difficulties, or ADHD.	4 weeks (4 times/week) [40 min]	AO + MI group: read an imagery script tailored to the task they were training (ADLs), then prompted to imagine the movement before watching a video of performing ADLs. (14) [Home environment]	Fine motor activity and then practice each ADL. (14)

Abbreviations: ADHD, Attention-Deficit/Hyperactivity Disorder; ADLs, Activities of Daily Living; AO, Action Observation; AO + MI, Action Observation plus Motor Imagery; BOTMP-2, Bruininks–Oseretsky Test of Motor Proficiency, Second Edition; CO-OP, Cognitive Orientation to Daily Occupational Performance; CT, Controlled Trial; Ctrl, Control; DCD, Developmental Coordination Disorder; DCDQ, Developmental Coordination Disorder Questionnaire; DSM-5, Diagnostic and Statistical Manual of Mental Disorders, Fifth Edition; IQ, Intelligence Quotient; MABC-2, Movement Assessment Battery for Children, Second Edition; MI, Motor Imagery; MIP, Motor Imagery and Planning; MP, Motor Planning; NCR, Not Clearly Reported; NDI, Neuromotor Development Index; PMOQ-T, Persian Motor Observation Questionnaire for Teachers; PMT, Perceptual Motor Training; RCT, Randomized Controlled Trial; TD, Typically Developing; VR, Virtual Reality. * 23 confirmed, 5 suspected. ** Once session performed clinical treatment, 4/weeks performed homework.

**Table 2:** Outcomes and results.

Author (Year) Country [Cit.]	Evaluation Tools; Timing (Follow-Up)	Statistical Approach	Results	Conclusions
Wilson (2016) Australia [16]	MABC; Pre-post (no f/u)	Pre-post change scores on MABC analyzed using a priori planned contrasts comparing intervention groups with wait-list control and MI vs. PMT; effect sizes (r) calculated; clinically meaningful change assessed using Smallest Detectable Difference.	Planned contrasts on total M-ABC scores showed an increase for MI and PMT (on average) with respect to the controls (*p* = 0.03; ES: MI r = 0.84, PMT r = 0.70); no difference between MI and PMT (*p* = 0.953); control group showed small effect (r = 0.14).	Both MI and PMT interventions significantly improved motor performance in children with DCD compared to controls, with similar large effect sizes, indicating that both approaches are effective.
Adams (2017) Netherlands [17]	MABC-2, DCDQ, MCQ; Pre-post (no f/u)	Descriptive statistics, including medians and individual scores; clinically meaningful change assessed via MABC-2 thresholds.	Individual Change Scores on the MABC-2 revealed that two children in the MI group and three children in the CO-OP group improved their total score with 2 or more standard scores, a change which is defined as the Smallest Detectable Difference (SDD 95%) and is regarded as clinically significant	Both MI and CO-OP training groups showed clinically significant improvement, suggesting the feasibility and perceived benefit of the MI protocol in children with DCD.
Bhoyroo (2019) Australia [18]	NDI, DCDQ, MP performance, MIP performance; During the test (no f/u)	Non-parametric tests (Mann–Whitney U for between-group, Wilcoxon Signed-Rank for within-group); effect sizes (r) calculated; Bonferroni corrections applied. Dependent variables (%ESC, %minimal rotation) computed per participant and condition.	Within-group analysis of the MI group showed significant increases in ESC and reduced minimal rotation strategy (*p* ≤ 0.014; ES r ≥ 0.66).	MI can effectively enhance motor planning in children with DCD, with significant and large improvements in ESC and reduced reliance on minimal rotation.
EbrahimiSani (2020) Iran [19]	The hand rotation task (MI), Anticipatory action planning test, Rapid online control test; Pre-post (2 months)	Repeated measures ANOVA and MANOVA on pre-test, post-test, and follow-up to assess effects of VR intervention on predictive modeling measures.	Between-group analyses revealed significant group × time interactions for MI (*p* = 0.039, η^2^ = 0.16) and action planning (*p* = 0.027, η^2^ = 0.17), with the experimental group outperforming the control group at post-test and follow-up. For online anticipatory action control, significant group × time interaction effects were found for total time on target (*p* < 0.001, η^2^ = 0.47), consecutive time on target (*p* < 0.001, η^2^ = 0.44), and distance from target (*p* = 0.001, η^2^ = 0.22), with improvements in the experimental group primarily from pre-test to post-test and maintained at follow-up. Within-group analyses showed significant improvements over time in the experimental group for MI (*p* = 0.003, η^2^ = 0.47) and action planning (*p* < 0.05, η^2^ = 0.65), whereas no significant changes were observed in the control group.	Motor imagery training within a VR-based intervention enhanced MI performance, action planning, and online anticipatory action control in children with DCD, producing lasting improvements in predictive motor control.
Marshall (2020) UK [20]	DCDQ, MABC-2 *, Completion time, Target-locking score, Movement kinematics; Pre-post (no f/u)	Mixed-design ANOVAs (Group × Time) for completion time, gaze control, path length, and normalized jerk; log transformations and Greenhouse-Geisser corrections applied when assumptions were violated; effect sizes as ηp^2^ reported.	Between-group analyses revealed significant group × time interactions, with the AO + MI group showing faster completion times at T2, T3 and post-test (*p* = 0.002–0.009) and higher target-locking scores from T1 through post-test (*p* ≤ 0.012) compared with the control group. Additionally, significant time effects were observed for total path length and normalized jerk in both groups (*p* < 0.001, ηp^2^ = 0.42; *p* < 0.001, ηp^2^ = 0.65, respectively).	AO + MI training significantly improved movement speed, visual attention, and movement smoothness in children with DCD, highlighting its effectiveness for enhancing motor performance.
Scott (2020) UK [21]	DCDQ (for allocation), MABC2; During the test (no f/u)	Three-factor mixed ANOVA for group (DCD vs. TD) × instruction (intentional imitation, MI, AO + MI) × habitual speed (fast vs. slow); independent *t*-tests for group differences within instruction; two-factor ANOVAs for instruction × speed within groups; Greenhouse-Geisser corrections applied; effect sizes ηp^2^ or Cohen’s d; LSD correction for pairwise comparisons.	Within-group analysis showed an improved imitation in the AO + MI group compared to MI alone (M = 127% vs. 116%, *p* = 0.021, ηp^2^ = 0.24) but not compared to simple imitation (*p* = 0.236). Habitual speed had no effect.	AO + MI clearly enhances action imitation in children with DCD. Within the experimental group, performance under AO + MI consistently exceeded that of MI alone, as reflected by higher cycle time ratios and a closer approximation to the performance of typically developing peers. These findings indicate that MI-based interventions can effectively support motor learning and action planning in children with DCD.
Scott (2023) UK [22]	MABC2, MIQ-C, DCDQ, Performance time, technique ratings for each task; Pre-post (2 weeks)	Mixed-effects models for continuous and ordinal outcomes with random intercepts and slopes; for subgroups with reduced data, mixed-measures ANOVAs with Tukey’s HSD post-hoc tests applied.	Between-group analysis showed a significant time × group interaction in the shoelace tying (*p* = 0.018, η^2^p = 0.22); post-test performance faster than controls (*p* = 0.045) and technique higher at post-test (*p* = 0.002) and retention (*p* = 0.011). Also cup stacking technique improved at post-test (*p* = 0.008) and retention (*p* = 0.01).	The AO + MI intervention effectively enhanced motor learning in children with DCD, producing significant improvements in both performance speed and movement technique across multiple daily tasks, with some effects maintained at retention. This supports the use of combined action observation and motor imagery to facilitate skill acquisition and motor planning in this population.

Abbreviations: AO, Action Observation; AO + MI, Action Observation plus Motor Imagery; CO-OP, Cognitive Orientation to Daily Occupational Performance; DCD, Developmental Coordination Disorder; DCDQ, Developmental Coordination Disorder Questionnaire; ESC, End-State-Comfort; f/u, Follow-up; MCQ, Motor Coordination Questionnaire; MABC, Movement Assessment Battery for Children; MABC-2, Movement Assessment Battery for Children, Second Edition; MI, Motor Imagery; MIQ-C, Motor Imagery Questionnaire-Children; MIP, Motor Imagery and Planning; MP, Motor Planning; NDI, Neuromotor Development Index; PMT, Perceptual Motor Training; SDD, Smallest Detectable Difference; TD, Typically Developing; VR, Virtual Reality. * Tool used for screening.

## Data Availability

No new data were created or analyzed in this study. Data sharing is not applicable to this article.

## Supplementary Materials

The following supporting information can be downloaded at: https://www.mdpi.com/article/10.3390/brainsci16020234/s1, Table S1: Search strategy; Figure S1: PRISMA flow-diagram.

## Abbreviations

The following abbreviations are used in this manuscript:

ADHD	Attention Deficit Hyperactivity Disorder
ADL/s	Activity/ies of daily living
AO	Action Observation
BOTMP-2	Bruininks–Oseretsky Test of Motor Proficiency Second Edition
CO-OP	Cognitive orientation to daily occupational performance
CT/s	Controlled Trial/s
Ctrl	Control
DCD	Developmental Coordination Disorder
DCDQ	Developmental Coordination Disorder Questionnaire
DSM-5	Diagnostic and Statistical Manual of Mental Disorders fifth edition
ESC	End-State Comfort
IMD	Internal Modeling Deficit
IQ	Intelligence Quotient
MABC-2	Movement Assessment Battery for Children Second Edition
MCQ	Motor Competence Questionnaire
MI	Motor Imagery
MIQ-C	Movement Imagery Questionnaire for Children
MP	Motor Planning
MIP	Motor Imagery and Planning
NCR	Not Clearly Reported
NDI	Neuromuscular Development Index
PMOQ-T	Persian Motor Observation Questionnaire for Teachers
PMT	Perceptual motor training
PRISMA	Preferred Reporting Items for Systematic reviews and Meta-Analyses
RCT/s	Randomized Controlled Trial/s
RoB2	Cochrane risk of bias tool version 2
ROBINS-I	Risk of Bias in Non-randomized Studies of Interventions
TD	Typically Developing
VR	Virtual Reality
WOS	Web of Science
