# Supplementary material for: Action Observation and Motor Imagery in Children with Developmental Coordination Disorder: A Systematic Review
Source: Brain Sci. 2026 Feb 17;16(2):234. doi: 10.3390/brainsci16020234 (PMC12938530; doi:10.3390/brainsci16020234)
Supplement: Supplementary file 1 [file brainsci-16-00234-s001.zip › brainsci-4103267-supplementary.pdf]

Table S1. Search strategy

Appendix A: search strategy

(((motor imagery) OR (action observation therapy) OR (mirror therapy)) AND (developmental coordination disorder)

((("motor"[All Fields] OR "motor s"[All Fields] OR "motoric"[All Fields] OR "motorically"[All Fields] OR "motorics"[All Fields] OR "motoring"[All Fields] OR "motorisation"[All Fields] OR "motorised"[All Fields] OR "motorization"[All Fields] OR "motorized"[All Fields] OR "motors"[All Fields]) AND ("imageries"[All Fields] OR "imagery, psychotherapy"[MeSH Terms] OR ("imagery"[All Fields] AND "psychotherapy"[All Fields]) OR "psychotherapy imagery"[All Fields] OR "imagery"[All Fields])) OR (("action"[All Fields] OR "action s"[All Fields] OR "actions"[All Fields]) AND ("observability"[All Fields] OR "observable"[All Fields] OR "observables"[All Fields] OR "observation"[MeSH Terms] OR "observation"[All Fields] OR "observe"[All Fields] OR "observed"[All Fields] OR "observer"[All Fields] OR "observer s"[All Fields] OR "observers"[All Fields] OR "observes"[All Fields] OR "observing"[All Fields] OR "watchful waiting"[MeSH Terms] OR ("watchful"[All Fields] AND "waiting"[All Fields]) OR "watchful waiting"[All Fields] OR "observations"[All Fields]) AND ("therapeutics"[MeSH Terms] OR "therapeutics"[All Fields] OR "therapies"[All Fields] OR "therapy"[MeSH Subheading] OR "therapy"[All Fields] OR "therapy s"[All Fields] OR "therapys"[All Fields])) OR ("mirror movement therapy"[MeSH Terms] OR ("mirror"[All Fields] AND "movement"[All Fields] AND "therapy"[All Fields]) OR "mirror movement therapy"[All Fields] OR ("mirror"[All Fields] AND "therapy"[All Fields]) OR "mirror therapy"[All Fields])) AND ("motor skills disorders"[MeSH Terms] OR ("motor"[All Fields] AND "skills"[All Fields] AND "disorders"[All Fields]) OR "motor skills disorders"[All Fields] OR ("developmental"[All Fields] AND "coordination"[All Fields] AND "disorder"[All Fields]) OR "developmental coordination disorder"[All Fields])

Translations

motor: "motor"[All Fields] OR "motor's"[All Fields] OR "motoric"[All Fields] OR "motorically"[All Fields] OR "motorics"[All Fields] OR "motoring"[All Fields] OR "motorisation"[All Fields] OR "motorised"[All Fields] OR "motorization"[All Fields] OR "motorized"[All Fields] OR "motors"[All Fields]

imagery: "imageries"[All Fields] OR "imagery, psychotherapy"[MeSH Terms] OR ("imagery"[All Fields] AND "psychotherapy"[All Fields]) OR "psychotherapy imagery"[All Fields] OR "imagery"[All Fields]

action: "action"[All Fields] OR "action's"[All Fields] OR "actions"[All Fields]

observation: "observability"[All Fields] OR "observable"[All Fields] OR "observables"[All Fields] OR "observation"[MeSH Terms] OR "observation"[All Fields] OR "observe"[All Fields] OR "observed"[All Fields] OR "observer"[All Fields] OR "observer's"[All Fields] OR "observers"[All Fields] OR "observes"[All Fields] OR "observing"[All Fields] OR "watchful waiting"[MeSH Terms] OR ("watchful"[All Fields] AND "waiting"[All Fields]) OR "watchful waiting"[All Fields] OR "observations"[All Fields]

therapy: "therapeutics"[MeSH Terms] OR "therapeutics"[All Fields] OR "therapies"[All Fields] OR

"therapy"[Subheading] OR "therapy"[All Fields] OR "therapy's"[All Fields] OR "therapys"[All Fields]

mirror therapy: "mirror movement therapy"[MeSH Terms] OR ("mirror"[All Fields] AND "movement"[All Fields] AND "therapy"[All Fields]) OR "mirror movement therapy"[All Fields] OR ("mirror"[All Fields] AND "therapy"[All Fields]) OR "mirror therapy"[All Fields]

developmental coordination disorder: "motor skills disorders"[MeSH Terms] OR ("motor"[All Fields] AND "skills"[All Fields] AND "disorders"[All Fields]) OR "motor skills disorders"[All Fields] OR ("developmental"[All Fields] AND "coordination"[All Fields] AND "disorder"[All Fields]) OR "developmental coordination disorder"[All Fields]

Figure S1. PRISMA flow-diagram

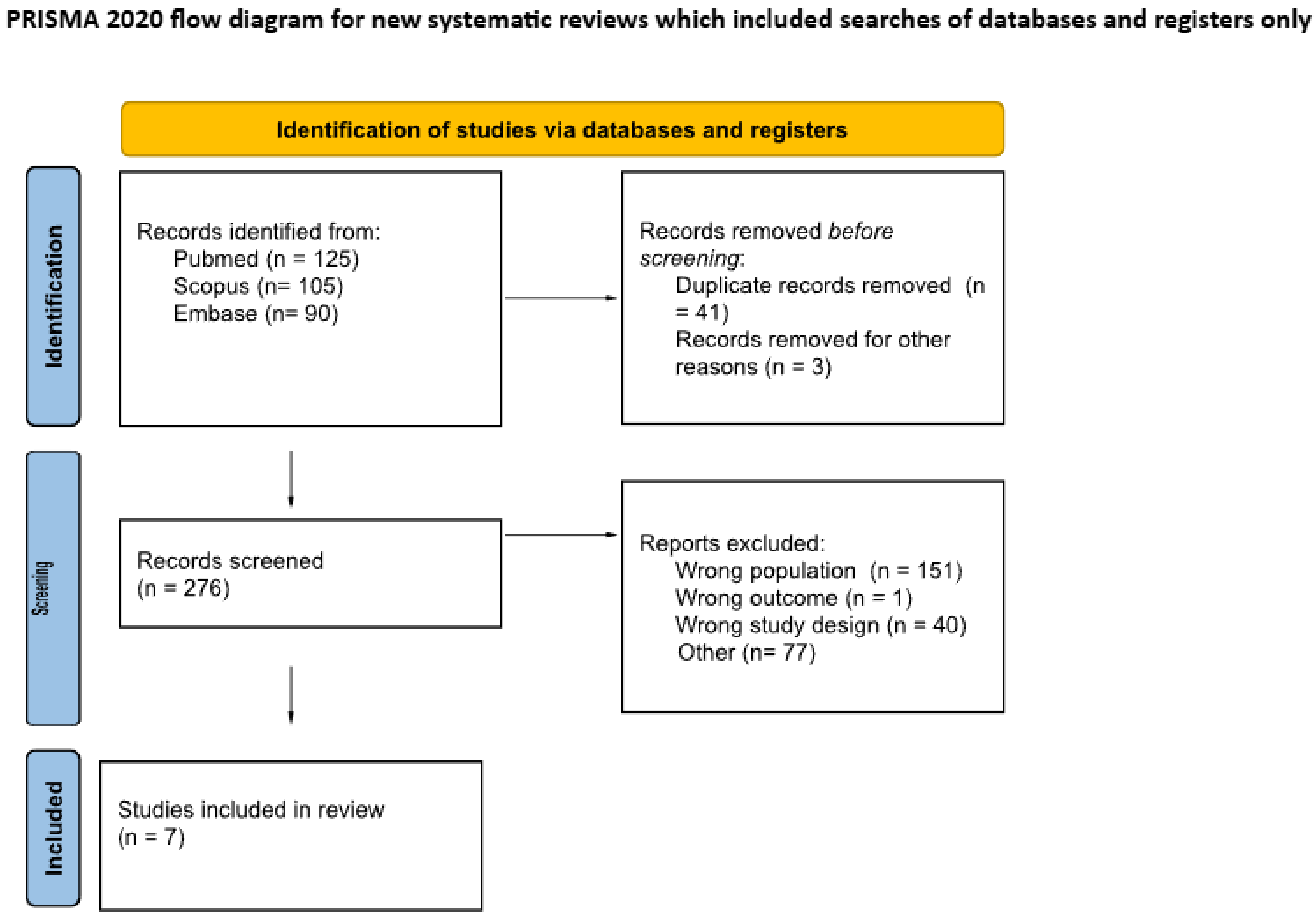

Figure S1: PRISMA flow-diagram. Source: Page MJ, et al. BMJ 2021;372:n71. doi: 10.1136/bmj.n71. This work is licensed under CC BY 4.0. To view a copy of this license, visit https://creativecommons.org/licenses/by/4.0/
